# Refining accuracy of RV–PA coupling in patients undergoing transcatheter tricuspid valve treatment

**DOI:** 10.1007/s00392-023-02339-5

**Published:** 2023-11-27

**Authors:** Atsushi Sugiura, Tetsu Tanaka, Refik Kavsur, Can Öztürk, Miriam Silaschi, Tadahiro Goto, Marcel Weber, Sebastian Zimmer, Georg Nickenig, Johanna Vogelhuber

**Affiliations:** 1 Heart Center Bonn, Department of Internal Medicine II, University Hospital Bonn, Venusberg-Campus 1, 53127 Bonn, Germany; 2 Heart Center Bonn, Department of Cardiac Surgery, University Hospital Bonn, Bonn, Germany; 3 Department of Clinical Epidemiology and Health Economics, School of Public Health, University of Tokyo, Tokyo, Japan

**Keywords:** Tricuspid regurgitation, RV–PA coupling, Echocardiography, Invasive measure, Outcomes

## Abstract

**Background:**

Assessing right ventricular (RV) function is paramount for risk stratification, which remains challenging in patients with tricuspid regurgitation (TR). We assessed RV–pulmonary artery (PA) coupling and its predictability of outcomes after transcatheter tricuspid valve repair (TTVR).

**Methods:**

Study participants comprised patients undergoing transcatheter tricuspid valve repair to treat symptomatic TR from June 2015 to July 2021. We calculated an RV–PA coupling ratio using a formula, which is dividing tricuspid annular plane systolic excursion (TAPSE) by echocardiographically estimated (ePASP) or invasively measured PASP (iPASP) at baseline. The primary outcome was all-cause mortality or heart failure rehospitalization within one year.

**Results:**

The study participants (n = 206) were at high surgical risk (EuroSCORE II: 7.4 ± 4.8%). The primary outcome occurred in 57 patients within one year. The c-statistics for the outcome were 0.565 (95% CI 0.488–0.643) for TAPSE/ePASP and 0.695 (95% CI 0.631–0.759) for TAPSE/iPASP. The correlation between the ePASP and iPASP was attenuated in patients with massive/torrential TR compared to those with severe TR (interaction p = 0.01). In the multivariable Cox proportional model, TAPSE/iPASP was inversely associated with the risk of the primary outcome (per 0.1-point increase: adjusted-HR 0.67, 95% CI 0.56–0.82, p < 0.001), independent of baseline demographics. According to the TAPSE/iPASP quartiles (i.e., ≤ 0.316; 0.317–0.407; 0.408–0.526; ≥ 0.527), the event-free survival was 43.4%, 48.3%, 77.9%, and 85.4% at one year after TTVR.

**Conclusion:**

RV–PA coupling predicts one-year mortality and heart failure rehospitalization after TTVR in patients with TR. The predictability is improved if invasively-measured PA pressure is included.

**Graphical abstract:**

Assessing right ventricular (RV) function is paramount for risk stratification. The present analysis confirms that RV–PA coupling, measured as TAPSE/PASP, predicts one-year mortality and heart failure rehospitalization in patients undergoing TTVR. There is a significant interaction between TR severity and the correlation of ePASP with iPASP, and therefore the correlation is attenuated in patients with massive to torrential TR. The predictability of RV–PA coupling is improved if PA pressure is measured invasively and included in the formula.

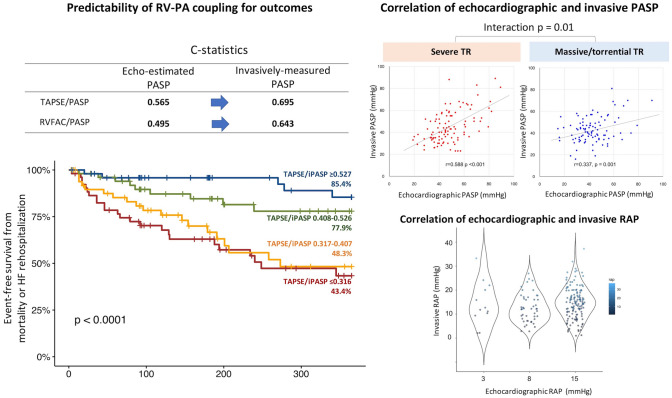

**Supplementary Information:**

The online version contains supplementary material available at 10.1007/s00392-023-02339-5.

## Introduction

Tricuspid regurgitation (TR) is associated with a dismal prognosis. TR can be treated less invasively through the use of recently developed transcatheter technologies [1, 2]. Assessing right ventricular (RV) function is paramount for risk stratification, which is, however, challenging in patients with TR. Although tricuspid annular plane systolic excursion (TAPSE) and RV fractional-area change (RVFAC) are common and easily obtainable parameters, these parameters are highly dependent on the volume status of the RV. Given that significant TR will lead to volume-overload and to pulmonary artery (PA) hypertension, the diagnostic accuracy for RV function using these parameters alone is limited.

RV–PA coupling seems more feasible for assessing RV function than other individual RV parameters, because it reflects the contractability of the RV under increased volume and afterload status in patients with TR. Brener et al. investigated RV–PA coupling, defined as the ratio between TAPSE and PA systolic pressure (PASP) as estimated by echocardiography, in patients undergoing transcatheter tricuspid valve treatment [3]. They reported that TAPSE/PASP was inversely associated with one-year mortality. However, echocardiography may miscalculate PASP in the presence of significant TR [4]. Instead, with the development of transcatheter interventions, a right-heart catheter has been increasingly recognized as a standard of care in the contemporary management of TR. Lurz et al. reported that invasively measured PASP (iPASP) may further discriminate clinical outcomes in patients undergoing TTVR beyond echocardiographically estimation of PASP [4]. Therefore, RV–PA coupling parameters using iPASP may provide a different cutoff value and better predictability of post-procedural outcomes than using echocardiographically estimated PASP (ePASP). This may be inducive in refining patient selection for TTVR in clinical practice.

In this context, we assessed RV–PA coupling parameters using ePASP or iPASP and compared them for outcome predictability.

## Methods

### Study populations

This is a retrospective analysis based on data from the Bonn registry, a prospective and consecutive data collection at the University Hospital of Bonn. All procedures were conducted in accordance with the Declaration of Helsinki and its amendments. Study participants comprised patients undergoing TTVR for the treatment of symptomatic TR from June 2015 to July 2021. All patients were deemed as being either ineligible or high risk for surgery. A standardized diagnostic workup was performed, including transesophageal echocardiography and left- and right-heart catheterization. In the present analysis, we included patients with sufficient information for the assessment of RV–PA coupling.

### Echocardiographic measurements

Doppler echocardiography was performed according to the current guidelines [5, 6]. TR severity was assessed using standard two-dimensional color Doppler methods integrating quantitative, semi-quantitative, and qualitative parameters according to the most recent guidelines. The TR jet was used to estimate TR peak gradient using the Bernoulli equation. The right atrial (RA) pressure was estimated by its size and collapsibility of the inferior vena cava. ePASP was then obtained by adding these two measures. TAPSE was measured as the peak excursion of the tricuspid annulus during the whole cardiac cycle in the apical four-chamber view. RV fractional area change (RVFAC) was calculated as (RV end-diastolic area − RV end-systolic area)/RV end-diastolic area × 100.

### RV–PA coupling parameters

We calculated an RV–PA coupling ratio by dividing TAPSE by PASP at baseline. ePASP and iPASP were applied to the equation as a PA component (i.e., TAPSE/ePASP, TAPSE/iPASP). We also assessed RVFAC/ePASP and RVFAC/iPASP in sensitivity analyses. iPASP was obtained through a right heart catheter at baseline.

### Outcome measures

The primary outcome was defined as a composite of mortality and rehospitalization due to heart failure within one year after TTVR. Each outcome was also assessed individually. We obtained clinical follow-up data through interviews at scheduled outpatient clinic visits, telephone interviews with their family, or documentation from the general practitioners.

### Patient and public involvement

Patients or the public were not involved in the design, or conduct, or reporting, or dissemination plans of this research.

### Statistical analysis

Continuous variables are reported as the mean ± standard deviation (SD) or medians with interquartile ranges (IQRs). T-tests are applied for normally-distributed variables and Mann–Whitney U test is conducted for non-normally distributed variables. Categorical variables are presented as numbers and percentages. Chi-square test is applied for the comparison. A test for Pearson’s correlation was conducted to assess the correlation of ePASP and iPASP, whereas a test for Spearman’s correlation was used for echocardiographic and invasive RA pressure measurements.

Given that the primary outcome is time-to-event data, we conducted a receiver operating characteristics (ROC) analysis using a Cox proportional model. The predictive accuracy of the outcome was evaluated using Harrell’s concordance index (Harrell’s C) [7, 8]. A smooth spline curve for hazard ratio (HR) of RV–PA coupling was depicted. The study participants were divided according to the quartiles of RV–PA coupling parameters. The Kaplan–Meier method was used for survival time analyses. A Cox proportional hazard model was conducted to assess the association of RV–PA coupling parameters with outcomes. Age, sex, EuroSCORE II, estimated glomerular filtration rate (eGFR), left ventricular (LV) ejection fraction, and chronic obstructive pulmonary disease were accounted in the model 1, while reduction in TR ≤ moderate at discharge was incorporated in the model 2 to adjust for the association [1, 3, 4, 9]. Furthermore, the association was tested among predefined subgroups (i.e., NYHA I/II or NYHA III/IV, LV ejection fraction ≤ 50% or > 50%, TR severity at baseline ≤ severe or ≥ massive, TR severity at discharge ≤ moderate or ≥ severe). All statistical analyses were performed using EZR version 1.37 (Saitama Medical Center, Jichi Medical University, Saitama, Japan), R version 3.5.2 (R Foundation for Statistical Computing), or IBM SPSS Statistics 26 (IBM Corporation, New York, USA).

## Results

### Study population

During the study period, a total of 206 patients met the inclusion criteria and were entered in the analysis. The mean age was 78.5 ± 7.1 years, and 58.3% were female. The study participants were at a high risk for surgery (EuroSCORE II: 9.0 ± 6.5%), and massive/torrential TR was observed in 100 (48.5%) of these patients. The majority of patients presented with atrial fibrillation (94.2%), preserved left ventricular ejection fraction (55.2 ± 10.1%), and a secondary etiology of TR (96.1%). All patients were treated either with transcatheter edge-to-edge repair (n = 185: 89.8%) or transcatheter annuloplasty (n = 21: 10.2%). No periprocedural death was observed. TTVR reduced the severity of TR (p < 0.001), with 78.0% of patients having TR 2+ or less at discharge.

### RV–PA coupling parameters

Variables regarding echocardiography and right heart catheterization are listed in Table 1. The median values of TAPSE, ePASP, and TAPSE/ePASP were 17.0 mm (IQR 15.0, 20.0 mm), 45.0 mmHg (IQR 36.3, 53.0 mmHg), and 0.392 mm/mmHg (IQR 0.287, 0.514 mm/mmHg). The median time from baseline echocardiography to invasive right heart catheterization was one day (IQR − 1, 5 days). The invasively-measured PASP (i.e., iPASP) was 42.0 mmHg (IQR 35.0, 52.0 mmHg), and the median of TAPSE/iPASP was 0.408 mm/mmHg (IQR 0.316, 0.526 mm/mmHg).

**Table 1:** Baseline characteristics

	All	1st quartile TAPSE/iPASP ≤ 0.316	2nd quartile TAPSE/iPASP 0.317–0.407	3rd quartile TAPSE/iPASP 0.408–0.526	4th quartile TAPSE/iPASP ≥ 0.527	p value
Age, years	78.4 ± 7.1	78.9 ± 7.4	77.7 ± 7.7	78.8 ± 6.2	78.5 ± 6.9	0.81
Sex female	26 (50.0)	26 (50.0)	23 (45.1)	39 (75.0)	32 (62.7)	0.009
NYHA class III or IV	42 (82.3)	42 (82.3)	46 (90.3)	44 (84.6)	48 (94.1)	0.25
Peripheral edema	37 (71.2)	37 (71.2)	30 (58.8)	25 (48.1)	24 (47.1)	0.046
EuroSCORE II, %	9.0 ± 6.5	10.8 ± 6.7	9.4 ± 7.6	8.5 ± 5.9	7.4 ± 5.2	0.052
Comorbidities						
Arterial hypertension	44 (84.6)	44 (84.6)	46 (90.2)	45 (86.5)	39 (76.5)	0.30
Diabetes	14 (26.9)	14 (26.9)	14 (27.5)	15 (28.8)	6 (11.8)	0.12
Dyslipidemia	32 (61.5)	32 (61.5)	32 (62.7)	26 (50.0)	18 (35.3)	0.019
COPD	38 (18.5)	12 (23.1)	10 (19.6)	7 (13.5)	9 (17.6)	0.64
Atrial fibrillation/flutter	49 (94.2)	49 (94.2)	47 (92.2)	46 (88.5)	46 (90.2)	0.78
Coronary artery disease	118 (57.3)	32 (61.5)	33 (64.7)	30 (57.7)	23 (45.1)	0.20
History of myocardial infarction	15 (28.8)	15 (28.8)	13 (25.5)	16 (30.8)	10 (19.6)	0.58
History of stroke	5 (9.6)	5 (9.6)	7 (13.7)	5(9.6)	7(13.7)	0.86
Prior PCI	20 (40.0)	20 (40.0)	13 (27.1)	16 (32.0)	11 (24.4)	0.38
Prior CABG	17 (32.7)	17 (32.7)	15 (29.4)	10 (19.2)	5 (9.8)	0.020
Prior pacemaker/ICD/C RT	21 (40.4)	21 (40.4)	15 (29.4)	13 (25.0)	15 (29.4)	0.39
Peripheral vascular disease	23 (44.2)	23 (44.2)	16 (31.4)	19 (36.5)	17 (33.3)	0.56
Laboratory data						
eGFR, ml/min/1.73 m^2^	49.6 ± 20.9	46.0 ± 20.9	52.4 ± 21.0	47.4 ± 20.1	52.3 ± 21.6	0.29
NT-pro-BNP, pg/ml	1906 (1150, 3995)	2556 (1599, 5148)	1798 (886, 3811)	1959 (1150, 3852)	1733 (1189, 3268)	0.08
Bilirubin, mg/dl	0.9 ± 0.6	1.0 ± 0.8	0.9 ± 0.5	0.8 ± 0.4	0.8 ± 0.4	0.12
Echocardiography						
LV ejection fraction, %	55.2 ± 10.1	52.8 ± 23.3	56.2 ± 10.4	54.8 ± 9.0	57.0 ± 8.1	0.17
LA volume, ml	88.6 ± 39.2	91.6 ± 34.3	91.7 ± 47.6	87.3 ± 39.7	83.6 ± 34.9	0.79
TR severity						0.58
Severe	34 (65.4)	34 (65.4)	24 (47.1)	27 (51.9)	25 49.0)	
Massive	14 (26.9)	14 (26.9)	21 (41.2)	21 (40.4)	21 (41.2)	
Torrential	4 (7.7)	4 (7.7)	6 (11.8)	4 (7.7)	5 (9.8)	
Etiology of TR						0.96
Primary	8 (3.9)	3 (5.8)	2 (3.9)	2 (3.8)	1 (2.0)	
Secondary	198 (96.1)	49 (94.2)	49 (96.1)	50 (96.2)	50 (98.0)	
Estimated PA systolic pressure	46.7 ± 14.8	53.8 ± 17.8	45.5 ± 14.0	43.9 ± 11.4	43.5 ± 13.0	< 0.001
TAPSE, mm	17.9 ± 5.2	13.4 ± 3.2	16.4 ± 3.3	18.5 ± 3.6	23.3 ± 4.6	< 0.001
RVFAC, %	43.2 ± 10.2	38.1 ± 8.2	41.6 ± 10.4	46.5 ± 10.4	47.1 ± 9.2	< 0.001
RA area, cm^2^	30.7 ± 9.7	29.8 ± 11.0	31.7 ± 10.3	31.1 ± 9.2	30.6 ± 8.6	0.87
Invasive measurement						
PCWP, mmHg	18.4 ± 6.8	22.5 ± 5.4	19.5 ± 6.6	16.8 ± 5.6	14.6 ± 6.7	< 0.001
PA systolic pressure, mmHg	44.2 ± 13.6	58.6 ± 13.7	45.8 ± 8.8	39.5 ± 7.8	32.9 ± 7.7	< 0.001
PA mean pressure, mmHg	27.9 ± 8.6	36.3 ± 8.5	28.4 ± 6.2	25.4 ± 5.5	21.2 ± 5.6	< 0.001
PA resistance, wood	2.39 ± 1.32	3.18 ± 1.59	2.21 ± 0.97	2.19 ± 1.31	1.99 ± 1.05	< 0.001
RA pressure, mmHg	14.0 ± 6.7	18.2 ± 6.6	14.3 ± 6.4	12.3 ± 6.0	10.9 ± 5.5	< 0.001
Medications						
Beta-blocker	165 (80.1)	44 (84.6)	39 (76.5)	42 (80.8)	40 (78.4)	0.74
ACE-I/ARB/ARNI	127 (61.7)	33 (63.5)	25 (49.0)	40 (76.9)	29 (56.9)	0.026
MRA	81 (39.3)	26 (50.0)	22 (43.1)	19 (35.6)	14 (27.5)	0.11
Dose of loop-diuretic	20.0 (10.0, 40.0)	27.5 (10.0, 40.0)	20.0 (10.0, 45.0)	20.0 (10.0, 40.0)	15.0 (10.0, 20.0)	0.053

*ACE-I* angiotensin-converting enzyme inhibitors, *ARB* angiotensin receptor blockers, *ARNI* angiotensin receptor/neprilysin inhibitor, *CABG* coronary artery bypass graft, *COPD* chronic obstructive pulmonary disease, *CRT* cardiac resynchronization therapy, *eGFR* estimated glomerular filtration ratio, *ICD* intracardiac defibrillator, *LA* left atrial, *LV* left ventricular, *MRA* mineralcorticoid receptor antagonist, *NT-pro-BNP* NT-pro-brain natriuretic peptide, *NYHA* New York Heart Association, *PA* pulmonary artery, *PCI* percutaneous coronary intervention, *PCWP* pulmonary capillary wedge pressure, *RA* right atrial, *RVFAC* right ventricular fractional area change, *TAPSE* tricuspid annular plane systolic excursion, *TR* tricuspid regurgitation

### Prediction for outcomes according to RV–PA coupling parameters

**Fig. 1:**
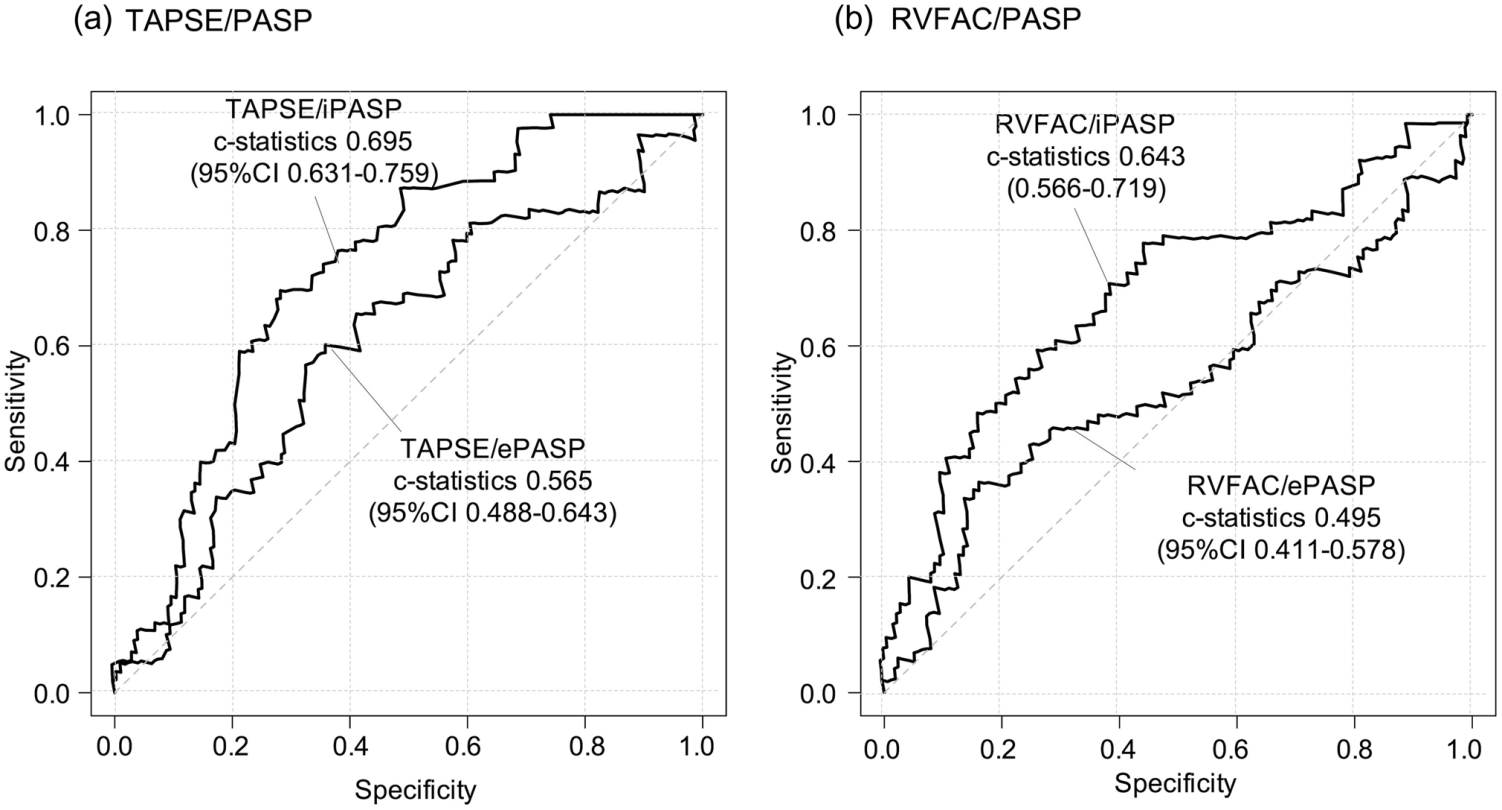
Receiver operating characteristics curves of RV–PA coupling. Shown are the receiver operating characteristics curves for each method of RV–PA coupling for predicting the outcome. Predictability was improved by measuring PA pressure invasively before applying the formulas

With the median follow-up duration of 201 days (IQR 98–424 days), 29 patients had died, and 40 patients had experienced rehospitalization due to heart failure, resulting in 57 cases of the primary outcome. The associations of right heart parameters with the primary outcome are listed in Supplemental Table 1. Compared to TAPSE/ePASP, TAPSE/iPASP showed better predictability for the primary outcome (Fig. 1A): the c-statistics was 0.565 (95% CI 0.488–0.643) for TAPSE/ePASP and increased to 0.695 (95% CI 0.631–0.759) when iPASP was applied to the formula (i.e., TAPSE/iPASP) (p < 0.001). The trend was consistent for RV–PA coupling using RVFAC (Fig. 1B). RVFAC itself was not associated with the primary outcome (Supplemental Table 1). Similarly, RVFAC/ePASP was not predictive for the outcome, while RVFAC/iPASP predicted the primary endpoint. As shown in Fig. 1, integrating the iPASP measurement led to a better prediction for the outcome (c-statistics: 0.495 for RVFAC/ePASP [95% CI 0.411–0.578]; c-statistics for RVFAC/iPASP: 0.643 [95% CI 0.566–0.719]).

In addition, we separately conducted ROC analyses in patients with severe and those with massive/torrential TR. TAPSE/iPASP showed a better discrimination irrespective of the TR severity, whereas the difference in Harrell’s C was numerically higher in patients with more advanced TR (delta c-statistics: 0.102 ± 0.035 in severe TR, p = 0.003; delta c-statistics: 0.179 ± 0.056 in massive/torrential TR, p = 0.001).

### Correlation between echocardiographic and invasive PA pressures

**Fig. 2:**
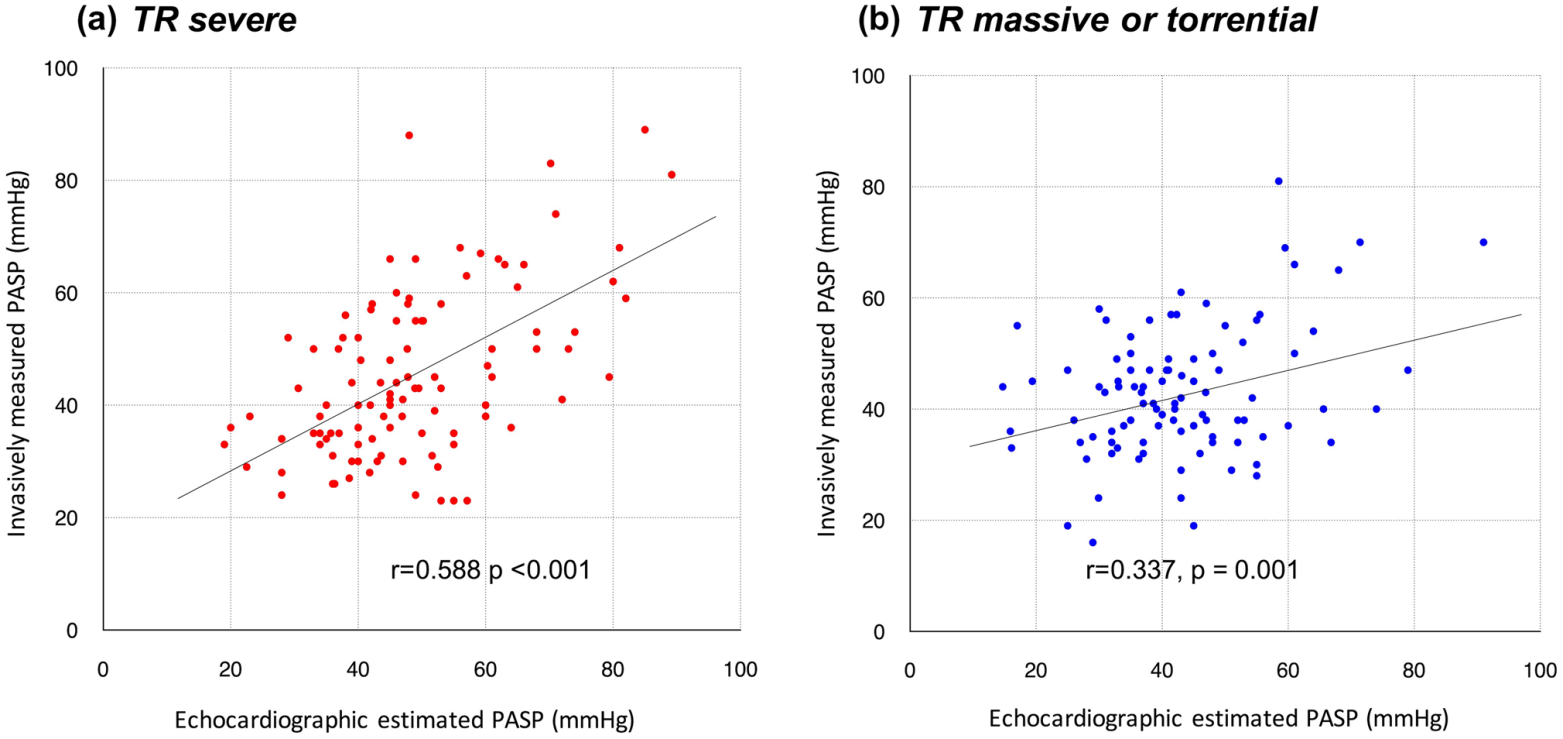
Correlation between echocardiographic estimated and invasively measured PASP. There was a significant interaction between TR severity and the accuracy of ePASP. The correlation between ePASP and iPASP was attenuated in patients with massive/torrential TR compared to patients with severe TR

There was a significant correlation between ePASP and iPASP in the total cohort (correlation coefficient 0.50, p < 0.001), whereas the correlation was attenuated in patients with massive to torrential TR (severe TR: correlation coefficient 0.588, p < 0.001; massive to torrential TR: correlation coefficient 0.337, p = 0.001; interaction p = 0.01) (Fig. 2). A similar trend was observed in the correlation between TR pressure gradient and iPASP (severe TR: correlation coefficient 0.546, p < 0.001; massive to torrential TR: correlation coefficient 0.331, p = 0.001; interaction p = 0.035).

**Fig. 3:**
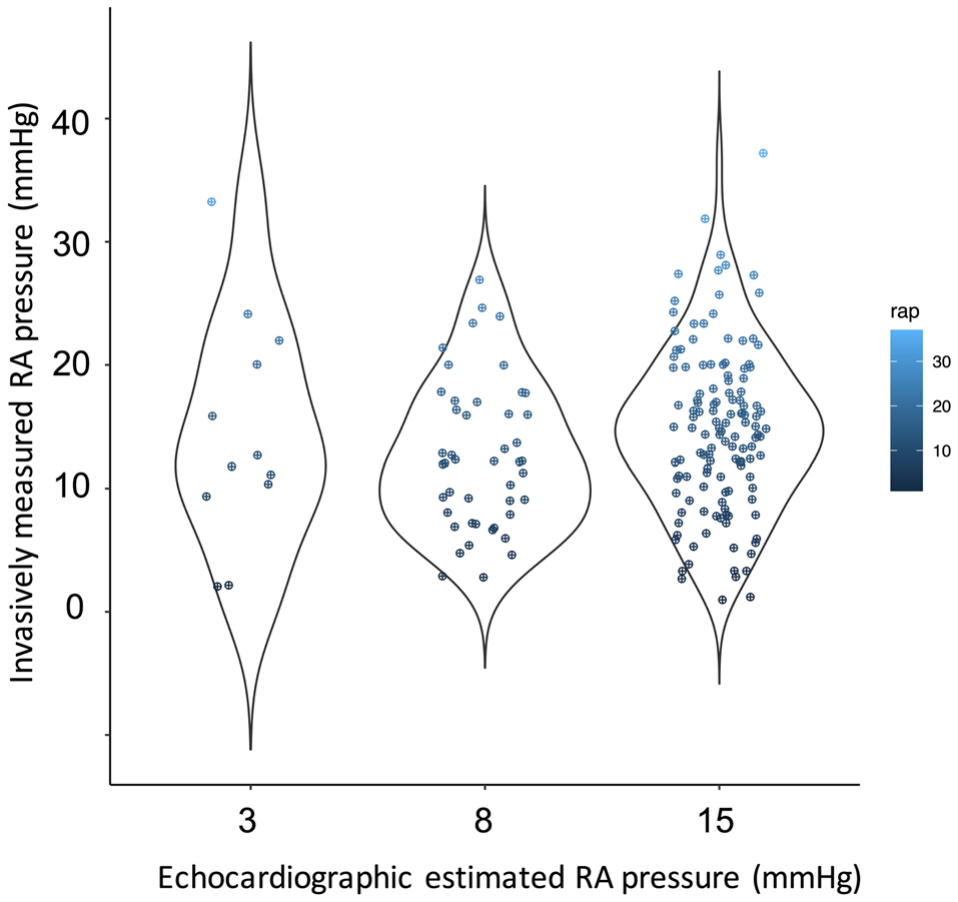
Correlation between echocardiographic and invasively measured RA pressure. The RA pressure assessed by echocardiography was weakly correlated with the invasive measurement (median 13 mmHg [IQR 9–18 mmHg]) (correlation coefficient 0.165, p value = 0.023). The median of the invasively-measured RA pressure was 12 mmHg (IQR 9–19 mmHg) in patients with echocardiographic RA pressure of 3 mmHg, 12 mmHg (IQR 8–16 mmHg) in those with echocardiographic RA pressure of 8 mmHg, and 15 mmHg (IQR 10–20 mmHg) in those with 15 mmHg of echocardiographic RA pressure

In addition, the echocardiographic RA pressure (median 15 mmHg [IQR 8–15 mmHg]) was modestly correlated with the invasive measurement (median 13 mmHg [IQR 9–18 mmHg]) (correlation coefficient 0.165, p value = 0.023) (Fig. 3). The RA pressure was likely to be underestimated in patients with the echocardiographic measurement of 3 and 8 mmHg. The miscalculation was also observed in patients with the echocardiographic RA pressure of 15 mmHg: a quarter of patients showed less than 10 mmHg of the invasive measurement, whereas another quarter showed more than 20 mmHg of the invasively-measured RA pressure.

### Clinical implication of TAPSE/iPASP

We divided the study participants according to TAPSE/iPASP (quartile 1: ≤ 0.316; quartile 2: 0.317–0.407, quartile 3: 0.408–0.526, quartile 4: ≥ 0.527). The baseline characteristics are listed in Table 1. Patients with lower TAPSE/iPASP were more likely to be male, having prior coronary artery bypass graft surgery, higher PA resistance and RA pressure, compared to patients with higher TAPSE/iPASP mm/mmHg. TR reduction to ≤ 2+ at discharge was consistently observed regardless of TAPSE/iPASP (Supplemental Table 2).

**Fig. 4:**
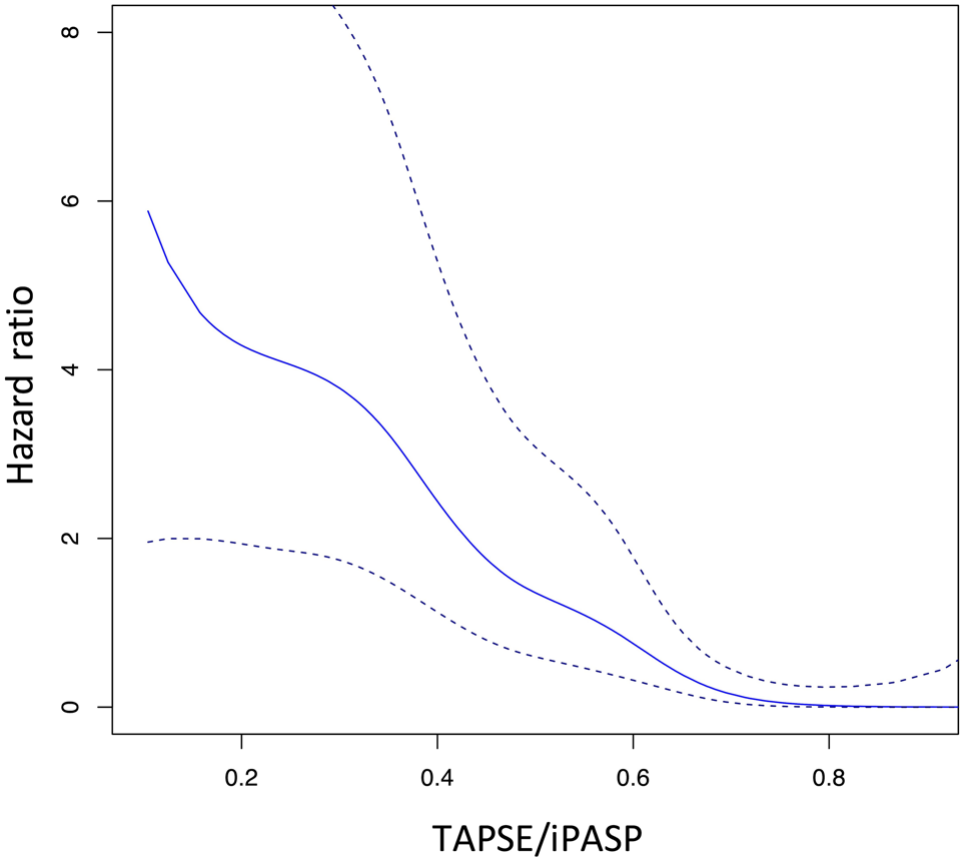
Smooth spline curve for association of TAPSE/iPASP with primary outcome. The spline curve depicts the linear association of TAPSE/iPASP with the primary endpoint

The spline curve depicts the linear association of TAPSE/iPASP with the primary endpoint (Fig. 4). TAPSE/iPASP was inversely associated with the primary outcome (per 0.1-point increase: adjusted-HR 0.67, 95% CI 0.56–0.82, p < 0.001) in the multivariable Cox proportional model (Table 2). Other parameters associated with the outcome were sex (male: adjusted-HR 2.26, 95% CI 1.21–4.20, p = 0.01) and eGFR (per 1 ml/min/1.73 m^2^ increase: adjusted-HR 0.98, 95% CI 0.97–0.99, p = 0.008).

**Table 2:** Cox proportional hazard model for primary endpoint

	Multivariable	
	Adjusted HR (95% CI )	p value
Model 1		
Age	0.98 (0.94–1.02)	0.36
Sex male	2.26 (1.21–4.20)	0.010
EuroSCORE II	0.99 (0.95–1.00)	0.07
COPD	1.54 (0.82–2.90)	0.18
eGFR	0.98 (0.97–0.99)	0.008
LV ejection fraction	0.98 (0.95–1.00)	0.07
TAPSE/iPASP per 0.1-point increase	0.67 (0.56–0.82)	< 0.001
Model 2		
Residual TR ≤ moderate at discharge	1.54 (0.86–2.75)	0.15
TAPSE/iPASP per 0.1-point increase	0.63 (0.52–0.76)	< 0.001

*eGFR* estimated glomerular filtration ratio, *COPD* chronic obstructive pulmonary disease, *LV* left ventricular, *PASP* pulmonary artery systolic pressure, *TAPSE* tricuspid annular plane systolic excursion

The association of TAPSE/iPASP with the outcome remained significant after adjusting for the TR reduction (Table 2). Also, the observed association was consistent among the predefined subgroups, including NYHA functional class, LV ejection fraction, the severity of TR at baseline, and the reduction in TR at discharge (Supplemental Fig. 1).

**Fig. 5:**
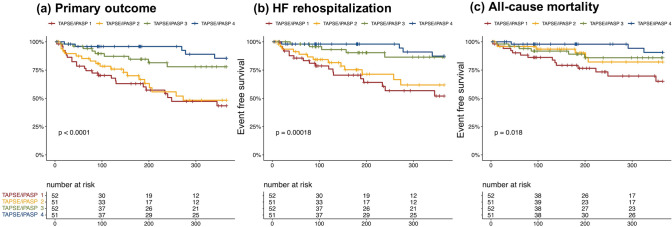
Survival analysis according to TAPSE/iPASP. According to the TAPSE/iPASP quartile (i.e., 1st quartile: ≤ 0.316; 2nd quartile: 0.317–0.407, 3rd quartile: 0.408–0.526, 4th quartile: ≥ 0.527), the event-free survivals were 43.4%, 48.3%, 77.9%, and 85.4% at one year after TTVR, respectively. Similar findings were observed for each outcome

Event-free survival curves were estimated and depicted using a Kaplan–Meier method (Fig. 5). According to the TAPSE/iPASP quarters (i.e., ≤ 0.316; 0.317–0.407; 0.408–0.526; ≥ 0.527), the event-free survivals were 43.4%, 48.3%, 77.9%, and 85.4% at one year after TTVR. Similar findings were observed for each outcome. The event-free survival from heart failure rehospitalization were 52.0%, 61.8%, 86.4%, and 87.3%, whereas survival from death were 65.0%, 82.1%, 85.9%, and 90.6% at one-year follow-up after TTVR.

## Discussion

This study assessed RV–PA coupling measured by the ratio of TAPSE and PASP in patients undergoing TTVR. The main findings can be summarized as follows (graphical abstract):

1. RV–PA coupling, measured as TAPSE/PASP, predicted adverse outcomes after TTVR in patients with TR.
2. The prediction for outcomes was improved if invasively measured PASP (i.e., iPASP) was applied to the formulas.
3. There was a significant interaction between TR severity and the correlation of ePASP with iPASP. The correlation was attenuated in patients with massive to torrential TR.
4. TAPSE/iPASP was associated with event-free survival after TTVR, independent of baseline demographics or TR reduction after TTVR.

According to current guidelines [5, 6], RV function is one of the main variables to weigh the indication of surgical or transcatheter tricuspid valve treatment in patients with TR. Despite the promising data and potential prognostic benefit of less invasive transcatheter technologies[2], concomitant RV dysfunction might pose challenges in the diagnosis and management of TR [4, 10]. Many TR patients receive appropriate clinical attention too late and appear with developed RV failure with multiple organ failures at the timing of interventions [9]. In the present study, patients represented a high surgical risk profile with the EuroSCORE II of 9.0 ± 6.5%. We found no periprocedural mortality, and TR reduction to 2+ or less was observed in 78% of patients, which is comparable to previous reports [11, 12]. Although an adequate reduction in TR may lead to an improved outcome [2, 13], the prognostic benefit of TR treatment may also be affected by concomitant RV function [14, 15]. Furthermore, TR reduction with TTVR might be linked to a risk of afterload mismatch of the RV after the procedure, especially in patients with inherent RV impairment [16].

RV–PA coupling is increasingly recognized as a more accurate parameter of RV systolic function, where RV parameters are indexed to a measurement of afterload (e.g., PASP) [15, 17]. The coupling of RV functional parameters (e.g., TAPSE, RVFAC) and PA pressure indicates that RV can compensate for an increased afterload of the pulmonary artery. The concept has recently widely been validated in patients with various types of diseases and also in patients with TR [3, 9–12]. Brener et al. reported that echocardiography-assessed RV–PA coupling (TAPSE/ePASP) was discriminative for one-year mortality in patients undergoing TTVR in a multicenter cohort [3]. Our findings are in line with the earlier studies and expand them by demonstrating better prediction for outcomes by applying TAPSE/iPASP (c-statistics 0.695) compared to TAPSE/ePASP (c-statistics 0.565). Furthermore, a similar association was observed in the RVFAC/ePASP and RVFAC/iPASP. The risk of outcomes was stratified by the TAPSE/iPASP. HF rehospitalization or all-cause mortality at one year were estimated in 56.6% for the first quartile of TAPSE/iPASP, whereas the event rate was 14.6% for patients in the fourth quartile of TAPSE/iPASP.

Echocardiography may miscalculate PASP in the presence of significant TR. The discordance between ePASP and iPASP was pronounced in patients with massive to torrential TR, inferring that PASP estimated by TR pressure gradient is likely underestimated because of a laminar flow of TR owing to a huge coaptation defect [4]. Lurz et al. reported that the diagnostic accuracy of ePASP to detect PA hypertension was only 55%. In the present study, the ePASP was somewhat higher than the iPASP, suggesting a possibility that the equation of ePASP itself may not be optimal in TR patients. Another explanation may be related to the estimation of RA pressure by echocardiography. An RA pressure estimation based on inferior vena cava diameters may not be correlated with the invasively measured RA pressure [18]. The diameter and shape of inferior vena cava are affected by RA pressure but are generally highly varied in individuals [19]. Assuming that patients with lower RV–PA coupling parameters are likely to have inferior outcomes even after treating TR, assessing PA and RA pressures will be critical. In line with the most recent European guideline [20], our findings underline that invasive cardiac catheterization is needed to directly assess right-heart pressures, thereby proceeding with clinical decision-making of TR [5].

### Limitations

Several limitations to our study should be acknowledged. First, the single-center, retrospective nature of this study, and the small sample size may limit the generalizability of the results. Nevertheless, the present study cohort represents data from one of the high-volume centers for TTVR. Second, no follow-up data on RV–PA coupling using iPASP was available. An early change in RV–PA coupling was reported in the most recent study, which needs to be validated in future studies using invasive measurements of right heart pressures. Also, factors associated with a change in RV–PA coupling following TTVR should be elucidated in further investigations. Third, the study cohort was regarded as one homogenous group, but we did not assess more detailed etiology of TR (i.e., atrial or ventricular secondary TR, cardiac implantable electronic device lead-induced TR). Fourth, since two different PASP values (echocardiographic and invasive) were measured at different times, patients might have been subjected to different hemodynamic influences. Finally, TAPSE reflects the longitudinal RV function but does not account for global RV function or might overestimate RV function in the presence of TR [10]. Nonetheless, the current analysis showed comparable c-statistics between the RV–PA coupling using TAPSE and RVFAC, which implies their feasibility in clinical practice for risk stratification in patients with TR. Magnetic resonance tomography might be more accurate for assessing function [10]. Nevertheless, impaired renal function or implanted pacemaker lead may limit its clinical use or feasibility of measurements. In contrast, TAPSE is the most commonly used and easily reproducible marker of RV function in clinical practice. Therefore, TAPSE/iPASP can be generalizable in each individual and each hospital.

## Conclusion

The present analysis confirmed that RV–PA coupling, measured as TAPSE/PASP, predicts one-year mortality and heart failure rehospitalization in patients undergoing TTVR. There was a significant interaction between TR severity and the correlation of ePASP with iPASP: the correlation was attenuated in patients with massive to torrential TR. The prediction for outcomes was improved if iPASP was measured and included in the formula. TAPSE/iPASP discriminated the event-free survival of patients with TR, independent of their baseline characteristics or TR reduction after TTVR.

### Supplementary Information

Below is the link to the electronic supplementary material.

Supplementary file1 Supplemental Figure 1. Association of TAPSE/iPASP with primary outcome according to predefined subgroups. (PDF 44 kb)

Supplementary file2 (DOCX 17 kb)

## Data Availability

The data underlying this article will be shared on reasonable request to the corresponding author.
