# Supplementary figures and images for: Refining accuracy of RV–PA coupling in patients undergoing transcatheter tricuspid valve treatment
Source: Clin Res Cardiol. 2023 Nov 27;113(1):177–86. doi: 10.1007/s00392-023-02339-5 (PMC10808486; doi:10.1007/s00392-023-02339-5)

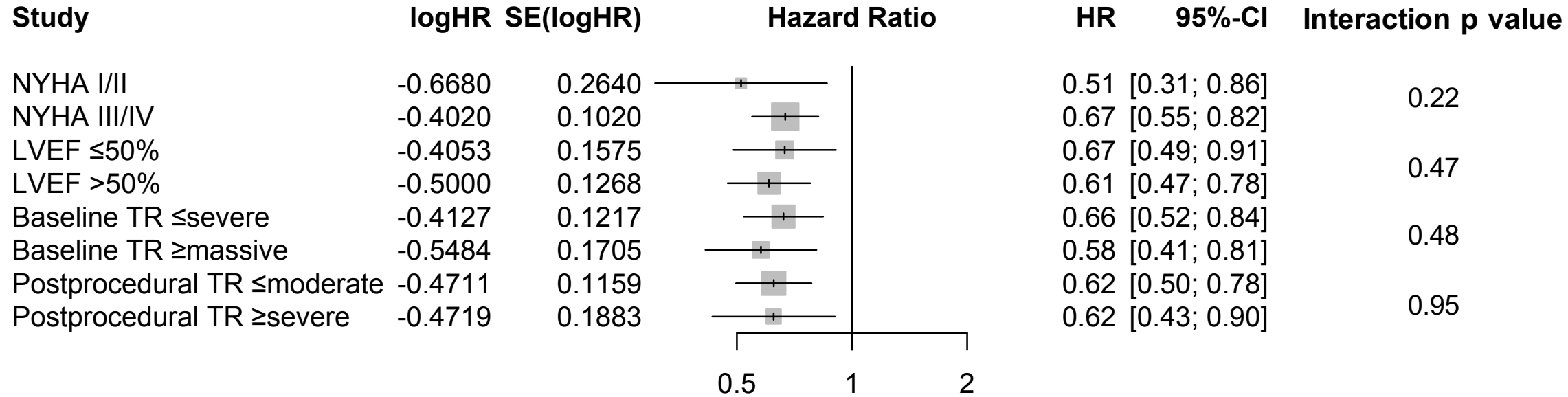

Supplement: Supplementary file 1 — Supplementary file1 Supplemental Figure 1. Association of TAPSE/iPASP with primary outcome according to predefined subgroups. (PDF 44 kb) [file 392_2023_2339_MOESM1_ESM.pdf]
