# Supplementary material for: Refining accuracy of RV–PA coupling in patients undergoing transcatheter tricuspid valve treatment
Source: Clin Res Cardiol. 2023 Nov 27;113(1):177–86. doi: 10.1007/s00392-023-02339-5 (PMC10808486; doi:10.1007/s00392-023-02339-5)
Supplement: Supplementary file 2 — Supplementary file2 (DOCX 17 kb) [file 392_2023_2339_MOESM2_ESM.docx]

**Supplemental Table 1. Association of right heart parameters with primary endpoint**

|  | Non-adjusted HR (95%CI) | p value |
| --- | --- | --- |
| TR massive/torrential | 0.79 (0.47-1.34) | 0.38 |
| TAPSE | 0.89 (0.84-0.95) | <0.001 |
| TAPSE / echocardiographic PASP per 0.1-point increase | 0.88 (0.75-1.03) | 0.12 |
| TAPSE / invasively measured PASP per 0.1-point increase | 0.63 (0.52-0.76) | <0.001 |
| RVFAC | 0.98 (0.95-1.00) | 0.07 |
| RVFAC / echocardiographic PASP per 0.1-point increase | 1.01 (0.96-1.07) | 0.62 |
| RVFAC / invasively measured PASP per 0.1-point increase | 0.89 (0.82-0.96) | 0.002 |
| Echocardiographic PASP | 1.00 (0.98-1.01) | 0.67 |
| Invasively measured PASP | 1.03 (1.01-1.05) | <0.001 |
| Echocardiographic RAP | 1.00 (0.93-1.07) | 0.93 |
| Invasively measured RAP | 1.09 (1.05-1.13) | <0.001 |

**Abbreviations**: PASP, pulmonary artery systolic pressure; RAP, right atrial pressure; TAPSE, tricuspid annular plane systolic excursion; TR, tricuspid regurgitation.

**Supplemental Table 2. Procedural outcomes according to TAPSE/iPASP groups**

|  | All | TAPSE/iPASP  ≤0.316 | TAPSE/iPASP  0.317–0.407 | TAPSE/iPASP  0.408–0.526 | TAPSE/iPASP  ≥0.527 | p value |
| --- | --- | --- | --- | --- | --- | --- |
| Device type intended to treat |  |  |  |  |  |  |
| MitraClip/TriClip/PASCAL | 185 (89.8) | 49 (94.2) | 45 (88.2) | 48 (92.3) | 43 (84.3) | 0.25 |
| Cardioband | 21 (10.2) | 3 (5.8) | 6 (11.8) | 4 (7.7) | 8 (15.7) | 0.046 |
| Conversion to surgery, n (%) | 0 | 0 | 0 | 0 | 0 | 0.99 |
| Post-procedural tricuspid valve gradient (mmHg) | 2.7±1.3 | 2.9±1.1 | 2.7±1.9 | 2.6±1.3 | 2.5±1.0 | 0.67 |
| Post-procedural TR grades |  |  |  |  |  | 0.52 |
| None or mild | 61 (29.8) | 12 (23.1) | 10 (19.6) | 17 (33.4) | 22 (43.1) |  |
| Moderate | 101 (49.3) | 28 (53.8) | 31 (60.8) | 23 (45.1) | 17 (33.3) |  |
| Severe | 37 (18.0) | 10 (19.2) | 7 (13.7) | 10 (19.6) | 10 (19.6) |  |
| Massive / torrential | 8 (3.9) | 2 (3.8) | 3 (5.9) | 1 (2.0) | 2 (4.0) |  |

**Abbreviations**: PASP, pulmonary artery systolic pressure; TAPSE, tricuspid annular plane systolic excursion
